# Knowledge and willingness of parents towards child girl HPV vaccination in Debre Tabor Town, Ethiopia: a community-based cross-sectional study

**DOI:** 10.1186/s12978-022-01444-4

**Published:** 2022-06-10

**Authors:** Gedefaye Nibret Mihretie, Tewachew Muche Liyeh, Alemu Degu Ayele, Habtamu Gebrehana Belay, Tigist Seid Yimer, Agernesh Dereje Miskr

**Affiliations:** Department of Midwifery, College of Medicine and Health Sciences, Debre Tabor University, Debre Tabor Town, Ethiopia

**Keywords:** Human papillomavirus vaccine, Knowledge, Attitude, Debre Tabor Town

## Abstract

**Background:**

Cervical cancer is currently the second-leading cause of cancer death among women in Ethiopia. Vaccination against the human papillomavirus (HPV) is an effective primary prevention strategy for HPV-related illnesses. The knowledge and willingness of parents toward the HPV vaccine are crucial to increasing the uptake of the vaccine. The vaccine's acceptance by children and young adolescents is dependent on parental consent. Therefore, this study aimed to assess knowledge, willingness, and associated factors of the human papillomavirus vaccine among parents of girls aged 9–14 years at Debre Tabor Town.

**Method:**

A community-based cross-sectional study was conducted among participants from December 10, 2020, to January 15, 2021. A simple random sample technique was used to include 638 participants. A structured face-to-face interviewer-administered questionnaire was used to collect data. The data were entered and analyzed using Epi-Data and SPSS software, respectively. Bivariate and multivariable analyses were used to examine the association. The Odds Ratio (OR), 95% CI, and p-values less than 0.05 were used to determine the statistical association.

**Results:**

Thirty-five percent (35.4%, 95% CI = 31.4%, 38.8%) and 44.8% (95% CI = 40.40%, 48.67%) of participants were knowledgeable about HPV vaccination and willing to get it, respectively. Being government employees (AOR = 5.46, 95% CI = 2.42, 9.34), and having a family history of sexually transmitted diseases (STD) (AOR = 1.76, 95% CI = 1.14, 2.72) were significantly associated with knowledge of the human papilloma virus (HPV) vaccine. Participants’ age (AOR = 1.43, 95% CI = 1.16, 2.87), secondary education and above (AOR = 1.70, 95% CI = 1.05, 2.74), fear of HPV infection (AOR = 2.29, 95% CI = 1.21, 4.32), and having good knowledge of the HPV vaccine (AOR = 3.30, 95% CI = 2.21, 4.93) were significantly associated with willingness to receive the HPV vaccine.

**Conclusion and recommendation:**

The knowledge and willingness of parents toward the HPV vaccine were low. Then, health officials should boost HPV vaccination promotion through public media. In schools, churches, mosques, and health facilities, health extension workers and health professionals provide information about the HPV vaccine for the parents. Mixed quantitative and qualitative studies are preferable for future research to address “why” issues.

## Plain language summary

Infection with the Human Papillomavirus (HPV) causes nearly 99% of cervical cancer cases and more than 20% of breast, neck, and anogenital cancers. The HPV vaccines protect against high-risk types of HPV (types 16 and 18), which account for approximately 70% of cervical cancers. Global coverage of the HPV vaccine was 39.7%, with high-income countries (68%), middle-income countries (28%), and lower-middle-income countries (2.7%). For different reasons, cervical cancer screening is very poor in Ethiopia (below 2%). Cervical cancer is mostly asymptomatic more than 20 years after infection. Primary prevention (enhancing the HPV vaccine) is the best way to protect women from cervical cancer. Adolescents’ uptake and acceptance of the vaccine depend on parental consent. Assessing parental knowledge and willingness at a community level is very crucial.

A simple random sample technique was used to include 638 participants. A structured and pre-tested face-to-face interviewer-administered questionnaire was used to collect the data. The data were entered and analyzed using Epi-Data, and SPSS software, respectively. Bivariate and multivariable analyses were used to examine the association.

Nearly one-third (35.4%) and less than half (44.8%) of participants were knowledgeable and willing to receive the HPV vaccination. The knowledge and willingness of the parents are significantly lower. Being government employees and having a family history of sexually transmitted diseases (STD) were factors affecting the knowledge of parents about the human papillomavirus vaccine. Participants’ age, secondary education and above, fear of HPV infection, and having good knowledge of the HPV vaccine were significantly associated with their willingness to use the HPV vaccine. The knowledge and willingness of the parents are significantly lower. Health officials and stakeholders should scale up HPV vaccine promotion through public media.

## Background

The Centers for Disease Control and Prevention states that almost 99% of cervical cancer cases and more than 20% of breast, neck, and anogenital cancers are caused by infection with human papillomavirus (HPV) [1]. In October 2011, the Advisory Committee on Immunization Services complied with guidelines to vaccinate all children, boys, and girls, to stop the ever-increasing incidence of HPV infection [2]. Currently, available HPV vaccines protect against high-risk types of HPV (types 16 and 18), which account for approximately 70% of cervical cancers and vaginal, oral, and anal cancers [3, 4]. A quadrivalent vaccine also protects against two low-risk types of the virus, which are responsible for 90% of genital warts (HPV types 6 and 11) [5–7].

Cervical cancer is the second most prevalent female cancer worldwide [8] and is the world's leading cause of female cancer mortality [9], especially in Sub-Saharan Africa [10]. Almost all of the girls who have been immunized against the HPV virus can be protected against more than 75% of cervical cancer cases [11]. In developed countries, cervical cancer has been decreasing for many years, largely due to the cervical cytology-screening program, which is now being replaced by HPV screening. However, cervical cancer is increasing in developing countries where nationwide cervical cancer screening is currently unavailable [12, 13].

Until recently, cytology-based screening programs were the main tool to detect and treat precancerous abnormalities and the early stages of cancer, preventing up to 80% of cervical cancers in developed countries. However, effective screening programs have been difficult to implement in low-resource settings. This is one reason why cervical cancer mortality rates are much higher in the developing world [14]. To prevent women from cervical cancer-related illness and mortality, the HPV vaccine is a better alternative than cytology screening or DNA testing, especially in resource-limited nations [15]. Global coverage of the HPV vaccine was 39.7%, with high-income countries (68%), middle-income countries (28%), and lower-middle-income countries (2.7%) [16]. The majority of cervical cancer researchers in Africa have focused on secondary prevention (cervical screening), whereas the number of publications focusing on primary prevention, notably HPV vaccination, is only approximately 23.4% [17].

Studies showed that in Hong Kong, 47.3%; Uganda, 56%; Southwest Nigeria, 79%; medical students in Southwest Ethiopia, 56.2% did not know about the HPV vaccine [18–21]. Socio-demographic factors like gender and educational level [22, 23], parents’ occupation [21, 24], participants’ family members’ history of cervical cancer, and participants who had information about the HPV vaccine (from school, newspaper, and internet) [24] and fear of HPV infection [25] were determinant factors of knowledge of HPV vaccine. In Southwest Nigeria, less than (40%); of Jima University medical students (36.8%); the United States (52%); in Morocco (27%) of the participant were willing to get the HPV vaccine for their adolescents [20–23], respectively. Parental age < 40 years [26], ethnicity [27], gender [28], parents who were worried about the potential risk of cervical cancer [29], parents’ adolescents who did not receive clinician recommendation to be vaccinated for HPV [30] were associated factors of the willingness of HPV vaccine.

Ethiopia is one of the resource-limited countries, access to cervical cancer screening is very less (below 2% among cervical screening eligible women) and human papillomavirus infection is mostly asymptomatic and causes cervical cancer mainly after 20 years. Because of quite challenging the natural history of HPV infection: (1) most sexually active individuals will acquire an HPV infection at some point in their lives. (2) A majority of HPV infections are asymptomatic and resolve spontaneously within a year or two. (3) HPV-related disease may not develop for years to decades following infection. Therefore, due to these challenges primary prevention (enhancing the HPV vaccine) is better than secondary prevention (HPV detection) to protect women from cervical cancer and genital wart.

Female adolescents’ uptake and acceptance of the vaccine are depending on parental consent. Ethiopia launched the HPV vaccine school-based approach implementation in December 2018. However, studies of knowledge and willingness toward the HPV vaccine have involved few Ethiopian parents, particularly in the study area, making it difficult to implement possible intervention strategies among this population. Therefore, assessing knowledge and willingness of human papillomavirus vaccine among parents of female adolescents and identifying factors affecting their children’s vaccine utilization is very vital in designing, implementing, and monitoring effective HPV vaccine immunization programs.

## Methods

### Study area and study period

A community-based cross-sectional study was conducted in Debre Tabor Town from January 1, 2021, to February 28, 2021. The town is located 665 km northwest of Addis Ababa (Ethiopia's capital city) and 97 km east of Bahir Dar City. The town is divided into six small administrative units called kebeles with a total population of 96,973 people, of whom 49,753 were men and 47,220 women, based on a population projection of Ethiopia for all regions at the Wereda level in 2017. All parents of girls aged 9–14 years who lived in Debre Tabor Town during the data collection period were included in the study.

Sample size determination: Epi-Info version 7 statistical software was used to calculate the sample size for objective one prevalence of knowledge (58.4%) and objective two prevalence of willingness to receive the HVP vaccine (59.9%) with the assumption of a 4% margin of error with a 95% confidence interval. Based on the assumptions, the final sample size was 641 and 634 with a 10% non-response rate for the prevalence of knowledge and prevalence of willingness, respectively.

### Sampling procedure

A simple random sampling technique was applied to select 641 parents for the study. Four thousand two hundred seventy households were in the town. Then, a census was conducted in all selected kebeles to identify parents who fulfilled the inclusion criteria (parents of girls aged 9–14). An identification number was given after a house-to-house visit. Then, a proportional-to-size allocation technique was employed to determine the study participants from each kebele. Finally, sample units were selected using a simple random sampling technique, and one mother and one father, or either of the mother or father per household, were interviewed. Participants in the selected household were not present at the time of data collection; three revisits were made to interview the mothers or fathers.

### Variables of the study

*Dependent variables* Parents’ knowledge and willingness to receive the HPV vaccine. Independent variables: socio-demographic variables (age, religion, marital status, educational status, occupation), reproductive health-related factors (family history of cervical cancer, fear of HPV infection, history of sexually transmitted diseases), sources of information (newspapers, radio, TV, schools, health professionals, health extension workers), understanding of the HPV vaccine, as well as HPV infection and cervical cancer).

### Operational definitions

*Knowledge of the HPV vaccine* Adolescents’ level of knowledge was measured based on correct responses to HPV vaccine knowledge questions. Each correct and incorrect response scores one (yes = 1) and zero (no and I do not know = 0) points, respectively. Using knowledge question items, participants who scored above or equal to 50% were considered to have knowledge (Yes), whereas those who scored less than the 50% score were measured as having no knowledge (No). Willingness is considered a participant’s score of 50% or above among the questions.

### Data collection procedure and techniques

A structured face-to-face interviewer-administered questionnaire was used to collect data. First, the questionnaires were developed in English and translated to the local language (Amharic) and then back to English by language experts to maintain consistency. Health extension workers and two Bachelors of Science (BSc.) midwives who are familiar with the local language and customs were recruited as data collectors and supervisors, respectively. The training was given to data collectors and supervisors for two days about data collection procedures, the content of the questionnaire, interview techniques, and confidentiality of the information obtained from the respondents.

### Data quality assurance

Data quality was ensured during collection, entry, and analysis. Before conducting the main study, a pretest was carried out on 32 (5%) of the sample. The principal investigator and supervisors conducted day-to-day on-site supervision during the whole period of data collection. At the end of each day, the questionnaires were reviewed and checked for completeness and accuracy by all the research team members, who undertook corrective discussion.

### Data processing and analysis

Epi-Data version 4.2 was used to code and enter the data, which was then exported to SPSS 23 for analysis. Descriptive analyses were conducted to summarize the data, and the results of the study were presented in the form of text, figures, and tables. Binary logistic regression analysis was performed by computing the odds ratio (OR) with a 95% confidence interval to see the crude association between each independent and dependent variable. Model fitness was checked by using Hosmer and Lemeshow goodness of fit. Finally, all independent variables by binary logistic regression p ≤ 0.2 were entered into multivariable logistic regression for further analysis, and significant associations were identified based on p < 0.05 and adjusted odds ratio (AOR) with 95% CI.

### Ethical clearance

Ethical clearance for this study was obtained from the ethical review committee of Debre Tabor University College of Medicine and Health Sciences. A supporting letter was obtained from Debre Tabor Town’s head office. Informed consent was obtained from participants after explaining the purpose of the study. Participants were informed that all the data obtained from them would be kept confidential and anonymous, and they had the right to withdraw at any point during data collection.

## Results

### Socio-economic characteristics of participants

Six hundred thirty-eight (638) parents were interviewed with a response rate of 99.5%. The mean age of the respondents was 36.41 (SD ± 5.8). Three hundred seventy-four (58.6%) of the parents were in the age group of 31–40. The majority of study participants, 574 (90.0%), were married, and 600 (94.0%) were Orthodox religious followers. Regarding educational status, more than half of the participants were in secondary school and above 387 (51.6%) (Table 1).

**Table 1:** Sociodemographic characteristics versus human papillomavirus vaccine among parents of children aged 9–14 Years in Debre Tabor Town, 2021

Variables	Frequency	Percent
Age		
23–30	51	8.0
31–40	374	58.6
41–46	213	33.4
Sex		
Male (fathers)	317	49.7
Female (mothers)	321	50.3
Marital status		
Single	21	3.3
Married	574	90.0
Widowed	15	2.4
Divorced	28	4.4
Religion		
Orthodox	600	94.0
Protestant /Muslim	38	6.0
Educational level		
Unable to read and write	52	8.2
Able to read and write	124	19.4
1–8th class	75	11.8
9–12th class	154	24.1
Diploma and above	233	36.5
Occupation		
Housewife	99	15.5
Self-employees	122	19.1
Government employees	227	35.6
Health professional	7	1.1
Merchants	183	28.7

### Reproductive related characteristics

Most of the study participants had no family history of cervical cancer 636 (99.7%). The majority of participants were afraid of sexually transmitted infections 586 (84.0%), and 25 (3.9%) participants had a history of sexually transmitted diseases (Table 2).

**Table 2:** Reproductive health versus human papillomavirus vaccine among parents of children aged 9–14 Years in Debre Tabor Town, 2021

Variables	Frequency	Percent
Heard about cervical cancer		
Yes	122	19.1
No	516	80.9
Family history of cervical cancer		
Yes	2	0.3
No	636	99.7
History of STD		
Yes	102	18.0
No	536	82.0
Fear of sexually transmitted infection		
Yes	536	84.0
No	102	16.0
Did your girl/s take the HPV vaccine		
Yes	87	13.6
No	551	86.4

### Sources of information about the HPV vaccine

**Fig. 1:**
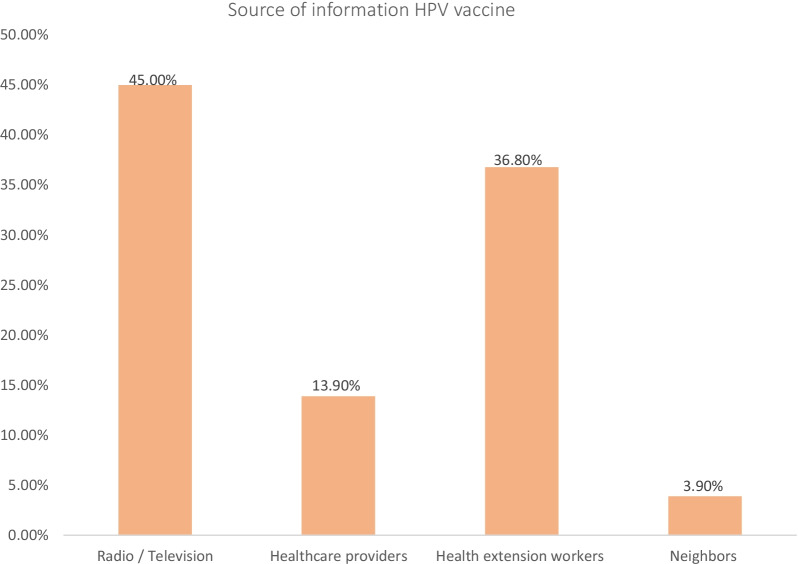
Source of information about human papillomavirus vaccine among parents of children aged 9–14 Years in Debre Tabor Town, 2021 (n = 311)

Nearly half of the participants 48.7% had heard of the HPV vaccine. Among these, below the half of the respondents said the main sources of information were radio/television 140 (45.0%) and health extension workers (36.8%), respectively (Fig. 1).

### Parents’ knowledge of the HPV vaccine and cervical cancer

Nearly one-third of participants (35.1%, 95% CI = 31.4%, 38.8%) had good knowledge about the human papillomavirus vaccine and cervical cancer. Of the 205 (32.1%) responded that the main cause of cervical cancer is HPV infection (Table 3).

**Table 3:** Knowledge of human papillomavirus vaccine and cervical cancer among parents of children aged 9–14 Years in Debre Tabor Town, 2021

Variables	Frequency	Percent
Having multiple sexual partners is the risk factor for HPV infection		
Yes	262	41.1
No	195	30.6
I don’t know	181	28.4
Sex at an early age increases the risk of transmission of HPV infection		
Yes	124	19.4
No	90	14.1
I don’t know	424	66.5
Being smokers increase the risk of HPV infection		
Yes	114	17.9
No	200	31.3
I don’t know	324	50.8
Sexual contact is the main transmitting route of HPV infection		
Yes	237	37.2
No	136	21.3
I don’t know	265	41.5
The main cause of cervical cancer is HPV infection		
Yes	205	32.1
No	174	27.3
I don’t know	259	40.6
People can transmit HPV to their partner even if they have no symptoms of infection		
Yes	118	18.5
No	246	38.6
I don’t know	274	42.9
Cervical cancer can be prevented by taking the HPV vaccine before sexual intercourse		
Yes	379	59.4
No	116	18.2
I don’t know	143	22.4
The recommended age for taking the HPV vaccine is 9–14-year-olds		
Yes	168	26.3
No	249	39.1
I don’t know	221	34.6
Knowledge		
Have knowledge (yes)	222	35.1
Have no knowledge (no)	414	64.9

### The willingness for the HPV vaccine

Two hundred eighty-six (44.8%, 95% CI = 40.40%, 48.67%) participants were willing to receive the HPV vaccination (Table 4).

**Table 4:** Willingness towards cervical cancer prevention and human papillomavirus vaccine among parents of children aged 9–14 Years in Debre Tabor Town, 2021

Variables	Frequency	Percent
A person who has only one sex partner can protect from HPV infection		
Strongly agree	221	34.6
Agree	197	30.9
Disagree	199	31.2
Strongly disagree	21	3.3
HPV vaccine education should be given for school adolescents		
Strongly agree	205	32.1
Agree	252	39.5
Disagree	116	18.2
Strongly disagree	21	3.3
Indifferent	44	6.9
Cervical cancer is a big problem for women		
Strongly agree	205	32.1
Agree	200	31.3
Disagree	170	26.6
Strongly disagree	42	6.6
Indifferent	21	3.3
Cervical cancer causes death in women		
Strongly agree	298	46.7
Agree	180	28.2
Disagree	118	18.5
Strongly disagree	21	3.3
Indifferent	21	3.3
Men involvement is important to prevent cervical cancer		
Strongly agree	103	16.1
Agree	241	37.8
Disagree	271	42.5
Strongly disagree	21	3.3
Indifferent	2	.3
Getting a Pap test examination is not an embarrassment		
Strongly agree	298	46.7
Agree	90	14.1
Disagree	208	32.6
Strongly disagree	21	3.3
Indifferent	21	3.3
Girls should get HPV vaccine before first sexual intercourse		
Strongly agree	336	52.7
Agree	183	28.7
Disagree	97	15.2
Strongly disagree	22	3.4
Health information about the HPV vaccine is needed for adolescents		
Strongly agree	218	34.2
Agree	197	30.9
Disagree	139	21.8
Strongly disagree	62	9.7
Indifferent	22	3.4
The HPV vaccine is effective to prevent cervical cancer		
Strongly agree	454	71.2
Agree	69	10.8
Disagree	115	18.0
Parents willingness		
Yes	286	44.8
No	352	55.2

### Why not have parents’ adolescent children receive the HPV vaccine?

**Fig. 2:**
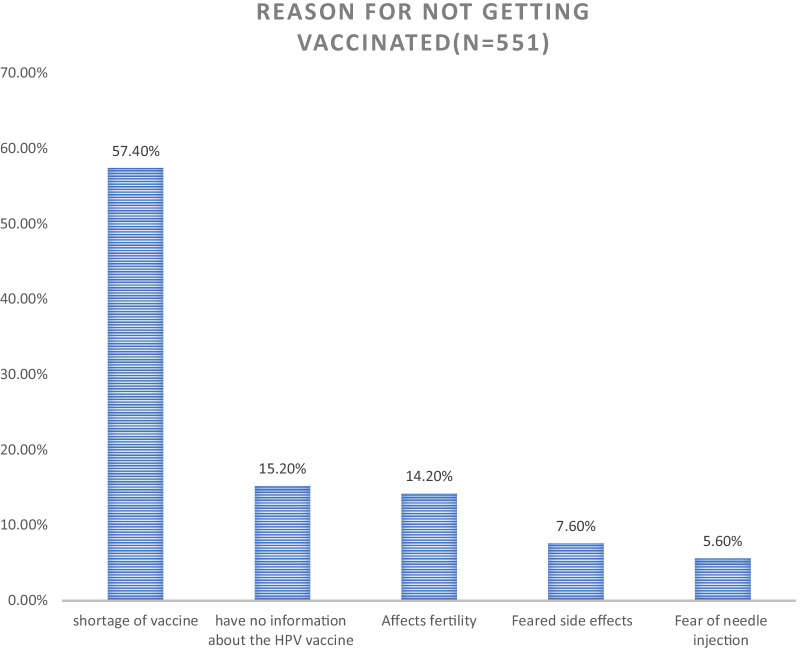
Reasons for not getting the human papillomavirus vaccine among parents of children aged 9–14 years in Debre Tabor Town, 2021 (n = 551)

The majority of parents’ girls (9–14 years old), 551 (86.4%), were not taking the HPV vaccine. Parents list various reasons for not taking the HPV vaccine (Fig. 2).

### Factors associated with knowledge of human papillomavirus vaccine and HPV vaccine

Two models were fitted to assess knowledge and attitude towards the HPV vaccine. The first model was fitted to assess the knowledge of the HPV vaccine. Variables such as occupation and family history of STDs were significantly associated with the knowledge of the HPV vaccine. Being government employees, they were 5.46 times more likely to know about the HPV vaccine (AOR = 5.46, 95% CI = 2.42, 9.34) as compared to those participants whose occupation was a housewife. Participants who had a family history of STDs were 1.76 times (AOR = 1.76, 95% CI = 1.14, 2.72) more likely to know about the HPV vaccine than those who had no history of STDs (Table 5).

**Table 5:** Factors associated with knowledge of human papillomavirus vaccine among parents of children aged 9–14 Years in Debre Tabor Town, 2021

Variables	Knowledge of HPV vaccine		COR (95% CI)	AOR (95% CI)	p-value
	Yes (N, %)	No (N, %)			
Sex					
Male	104 (46.4)	213 (51.5)	0.81 (0.59, 1.13)	0.80 (0.58, 1.12)	
Female	120 (53.6)	201 (48.5)	1		
Educational status					
No formal education	72 (32.1)	104 (25.1)	1		
Primary education	77 (34.4)	150 (36.3)	0.74 (0.49, 1.11)	0.76 (0.50, 1.15)	
Secondary and above	75 (33.5)	160 (38.6)	0.67 (0.45, 1.01)	0.70 (0.46, 1.05)	
Occupation					
Housewife	18 (8.1)	92 (22.2)	1		0.001
Government employees	60 (26.8)	63 (15.2)	4.86 (2.62, 9.01)	5.46 (2.42, 9.34**	
Self-employees	31 (13.8)	61 (14.7)	2.59 (1.33, 5.05)	2.57 (1.25, 5.27)	
Merchants	69 (30.8)	114 (27.5)	3.09 (1.72, 5.56)	3.10 (1.62, 5.93)	
Family history of STD					
Yes	48 (21.4)	54 (13.0)	1.81 (1.18, 2.79)	1.76 (1.14, 2.72)**	0.002
No	176 (78.6)	360 (89.0)	1		

**Variables were significant at p-values of < 0.05

### Factors associated with willingness of human papillomavirus vaccine and cervical cancer

The second model was fitted to assess factors associated with the attitude toward the HPV vaccine among parents of children aged 9–14 years. Variables such as the age of participants, educational status, fear of HPV infection, and knowledge of the HPV vaccine were significantly associated with the attitude of parents of children aged 9–14 years towards the HPV vaccine. Participants whose age was 31–40 years old were 1.43 times (AOR = 1.43, 95% CI = 1.16, 2.87) more likely to have willingness towards HPV vaccine utilization as compared to participants whose age was greater than or equal to 41 years old.

Participants who had secondary education and above were 1.7 times (AOR = 1.70, 95% CI = 1.05, 2.74) more likely to have willingness toward the HPV vaccine, as compared to those participants who had no formal education (unable to read and write plus able to read and write). Participants who fear HPV infection were 2.29 times (AOR = 2.29, 95% CI = 1.21, 4.32) more likely to have willingness toward the HPV vaccine as compared to participants who did not fear HPV infection. Participants who knew about the HPV vaccine and cervical cancer were 3.30 times (AOR = 3.30, 95% CI = 2.21, 4.93) more likely to have willingness toward the HPV vaccine as compared to those participants who did not know (Table 6).

**Table 6:** Factors associated with the attitude of human papillomavirus vaccine and cervical cancer among parents of children aged 9–14 Years in Debre Tabor Town, 2021

Variables	Willingness towards HPV vaccine		COR (95% CI)	AOR (95% CI)	p-values
	Yes (N, %)	No (N, %)			
Age					
23–30	12 (6.7)	39 (8.5)	1.08 (0.52, 2.24)	0.78 (0.33, 1.85)	0.003
31–40	119 (66.9)	255(55.4)	1.64 (1.11, 2.43)	1.43 (1.16, 2.87)**	
41–46	47 (26.4)	166(36.1)	1	1	
Sex					
Male	103 (57.9)	214 (46.5)	1.57 (1.11, 2.23)	1.49 (1.00, 2.22)	
Female	75 (42.1)	246 (53.5)	1	1	
Educational status					
No formal education	33 (18.5)	143 (31.1)	1		0.001
Primary education	77 (43.3)	150 (32.6)	2.22 (1.39, 3.55)	1.15 (0.34, 3.44)	
Secondary and above	68 (38.2)	167 (36.3)	1.76 (1.10, 2.82)	1.70 (1.05, 2.74)**	
Occupation					
Government employees	64 (36.0)	163 (35.4)	1		
Others*	114 (64.0)	297 (64.6)	1.02 (0.71, 1.46)	1.02 (0.51, 2.01)	
Fear of HPV infection					
Yes	162 (91.0)	374 (81.3)	2.32 (1.32, 4.09)	2.29 (1.21, 4.32)**	0.001
No	16 (9.0)	86 (18.7)	1		
Did your child took HPV vaccine?					
Yes	118 (66.3)	320 (70.0)	1		
No	60 (33.7)	140 (30.0)	0.86 (0.59, 1.24)	0.65 (0.43, 1.00)	
Knowledge on cervical cancer and HPV vaccine					
No	115 (49.4)	301 (82.8)	1		0.001
Yes	153(50.6)	69 (17.2)	5.80 (3.37, 7.61)	3.30 (2.21, 4.93)**	

*Self-employ, farmer, merchant, daily labourer

**Variables were significant at p-values of < 0.05

## Discussion

This study was conducted to assess knowledge and willingness of the HPV vaccine and associated factors among parents of children aged 9–14 years in Debre Tabor Town, Ethiopia. The involvement of parents in the decision to take the HPV vaccine for their children is very crucial for the acceptability and utilization of the vaccine. However, different factors such as participant age, educational status, occupation, family history of STD, and fear of HPV infection were significantly associated with knowledge and willingness to use the HPV vaccine.

Of the participants who had had information about the HPV infection and vaccine (48.7%), the two most important sources were radio/television (45.00%) and health extension workers (36.8%), followed by health care providers. More than half of the study participants (51.3%) had no information about the vaccine. Parents had more information about the immunization as their daughters had received the HPV vaccine. Fathers have access to more information than mothers do. This might be because parents gain access to information about the vaccine from their children after vaccination, and mothers, mostly because they are homemakers.

The prevalence of knowledge of parents about the HPV vaccine was 35.1%, which was in line with the systematic review and meta-analysis study (37%) [29], Nigeria (36.5%) [31], but lower than studies conducted in Romania (85.8%) [32], the United Kingdom (54.8%) [33], Kenya (48%) [34], and Thailand (60%) [28]. The explanation might be a lack of HPV vaccination advertising in the media or on social media. In contrast to these findings, other studies conducted among health professionals in Lagos in Nigeria [35], Enugu in Nigeria [36], and South Africa [37] revealed comparatively high levels of knowledge of 85.0%, 74.0%, and 96.0%, respectively. The variations in awareness in these earlier studies are most probably related to the health personnel's greater exposure to information regarding HPV infection in the health facility. On the other hand, this finding was higher than a study conducted in Iran (24%) [26]. This variation might be due to differences in the study setting, study population, and time of the study.

Forty-four percent (44.4%) of participants had a willingness to the HPV vaccine and cervical cancer prevention. This study was lower than the study conducted on Danish parents (80%) [38], Canada (70%) [39], Nigeria (81.8%) [31], Kenya (89%) [34], Tanzania (93.0%) [40], Honduras (91.0%) [41], and Birmingham (88.1%) [27]. This might be explained by a lack of understanding about the advantage of the HPV vaccination, fear of adverse effects, and concern about infertility as a result of the vaccination.

The reasons were given by those respondents why their children were not getting the human papillomavirus vaccine. The major reasons for not immunizing the HPV vaccine were the scarcity/cost of the HPV vaccine 57.4%. The second most reason was poor information about the HPV vaccine 15.2%, having doubts about HPV vaccination (undesirable impact on fertility) 14.2%, feared side effects 7.6%, and fear of needle injection 5.6%. This was similar to a study conducted in Nigeria were high cost (55.6%), worries about the side effects (48.1%), and poor availability (25.9%) of the vaccines were the main factors [31].

In this study, the odds of knowing the HPV vaccine were higher among parents of girls who worked as government employees as compared to participants whose occupations were housewives. This finding was in line with studies conducted in Saudi Arabia [24]. Participants who had relatively high educational status, agree to take the vaccine [32] and government employees are educated. Participants that are more educated may have better access to media (print, social, and mass media) exposure to HPV vaccination information. Participants who had family exposure to sexually transmitted diseases were more likely to know about the vaccine than those who did not have exposure to sexually transmitted diseases (STDs). This might be explained by the exposure to STDs in the family, health facility visits, and the gaining of important information, including the HPV vaccine.

Participants whose age was 31–40 years old were more likely to have a willingness as compared to those participants whose age was greater than or equal to 40 years old. This finding was similar to a study conducted in Iran [26]. The reason is unclear, but it is claimed that vaccines are our country’s most recent innovation, and young adults may have more information than older adults may have. Educational status was also significantly associated with a willingness to the HPV vaccine. Participants who had secondary education or above were more likely to have a willingness as compared to participants with no formal education. This might be because parents who have secondary and above-secondary educational levels are more likely to have information from school, mass media, newspapers, and the internet.

Parents who fear the infection of the human papillomavirus vaccine were more likely to experience a willingness to take the HPV vaccine than parents who did not fear the infection of HPV. These might be parents who have a fear of acquiring an HPV infection; they might have an intention to understand the prevention of the HPV infection.

Parents’ knowledge about cervical cancer and the HPV vaccine was significantly associated with their willingness to receive the HPV vaccine. Parents who knew were three times more likely to have a willingness to have the HPV vaccine as compared to those parents who did not know. This was similar with others studies in North Gondar [42], Malaysia [43, 44], Honduras [41], United Arab Emirate [45], Europe [46], and Kenya [47].This might be explained by the parents who have evidence about the route of transmission, a consequence of infection, and complications of cervical cancer that forced them to agree to take the HPV vaccine.

### Strength and limitation

Community-based study design and using a large sample size can be taken as the strength of the study. The limitation would be due to recall bias that might be faced. Another limitation of this study focused on the quantitative approach, which could not address the “why” questions in detail.

## Conclusion and recommendation

In this study, the knowledge and willingness of parents toward the HPV vaccine were low. Then, health authorities through mass media should strengthen HPV vaccine promotion. Health extension workers and health professionals provide pertinent information about the vaccine in schools, and health facilities by delivering leaflets and brochures. Mixed quantitative and qualitative studies are better for further investigations to answer “why” questions.

## Data Availability

All data were available within the manuscript.

## Abbreviations

AOR: Adjusted odds ratio
COR: Crude odds ratio
HPV: Human papillomavirus
STD: Sexually transmitted disease
WHO: World health organization
